# Can Walking or Biking to Work Really Make a Difference? Compact Development, Observed Commuter Choice and Body Mass Index

**DOI:** 10.1371/journal.pone.0130903

**Published:** 2015-07-08

**Authors:** Timothy R. Wojan, Karen S. Hamrick

**Affiliations:** U.S. Department of Agriculture Economic Research Service, Washington, DC, United States of America; Old Dominion University, UNITED STATES

## Abstract

**Objectives:**

Promoting active commuting is viewed as one strategy to increase physical activity and improve the energy balance of more sedentary individuals thereby improving health outcomes. However, the potential effectiveness of promotion policies may be seriously undermined by the endogenous choice of commute mode. Policy to promote active commuting will be most effective if it can be demonstrated that 1) those in compact cities do not necessarily have a preference for more physical activity, and 2) that current active commuting is not explained by unobserved characteristics that may be the true source of a lower body mass index (BMI).

**Methods:**

Daily time-use diaries are used in combination with geographical characteristics of where respondents live and work to test 1) whether residents of more compact settlements are characterized by higher activity levels; and 2) whether residents of more compact settlements are more likely to bike or walk to work. An endogenous treatment model of active commuting allows testing whether reductions in BMI associated with walking or biking to work are in fact attributable to that activity or are more strongly associated with unobserved characteristics of these active commuters.

**Results:**

The analysis of general activity levels confirms that residents of more compact cities do not expend more energy than residents of more sprawling cities, indicating that those in compact cities do not necessarily have a preference for more physical activity. The endogenous treatment model is consistent with walking or biking to work having an independent effect on BMI, as unobserved factors that contribute to a higher likelihood of active commuting are not associated with lower BMI.

**Conclusions:**

Despite evidence that more compact settlement patterns enable active commuting, only a small share of workers in these areas choose to walk or bike to work. In general, the activity level of residents in more compact cities and residents in more sprawling areas is very similar. But, there is a robust association between active commuting and lower body mass index that is not explained by unobserved attributes or preferences suggests that policies to promote active commuting may be effective. In particular, active commuting has a greater effect on BMI. Consequently, compact settlement appears to be an effective infrastructure for promoting more active lifestyles. The policy challenge is finding ways to ensure that this infrastructure is more widely utilized.

## Introduction

Lower body mass index (BMI) observed in more dense settlements has resulted in a large body of research examining the effect of urban form on health outcomes [1–5]. Since compact settlements are more walkable and sometimes discourage car ownership, it is reasonable to assume that a major driver of the BMI-compactness association is higher levels of physical activity (PA). However, mixed empirical results from studies that have explicitly examined the relationship between physical activity and compact development question the causes behind this association [6–9]. The efficacy of more compact development as a policy response to the obesity epidemic is further questioned by possible behavioral explanations of the phenomenon: if people who are predisposed to higher physical activity levels prefer to live in more compact settlements, then greater availability of compact development may have no effect on those predisposed to a more sedentary lifestyle [10,11].

This paper endeavors to advance the obesity/compact settlement debate in the U.S. in three ways: 1) it explicitly examines the physical activity/compact settlement association using the American Time Use Survey that adds a data point—albeit one characterized by extensive coverage—to the collection of mixed findings; 2) it explicitly examines the association between compact development and walking or biking to work, which may be regarded as an archetypal contribution of compact settlement to physical activity; and 3) it explicitly examines the association between walking or biking to work and BMI, after controlling for the endogeneity of commute mode choice. Our findings support the argument that compact development by itself does not induce greater physical activity but instead plays the traditional role of infrastructure as a possible enabler of preferred actions, in this case, more active lifestyles [12]. An endogenous treatment model confirms that the substantial reduction in BMI associated with walking or biking to work—which is still relatively rare among commuters—is not attributable to unobserved factors. Thus, policy to promote active commuting more widely may be effective in helping to stem the obesity epidemic.

## Background

French et al. [13] reviewed the literature on the environmental influences on eating and physical activity. Among their findings was that “[a] tremendous potential exists for increasing the population’s physical activity by making environmental changes that would encourage and support the use of walking and bicycling as a mode of transportation.” (pp. 324–5) They also found that simple prompts to use stairs, such as small signs, can be effective in increasing individuals’ activity levels. Likewise, Owen et al. [14] found “…consistent evidence over the past decade for the built environment as a significant correlate of physical activity (p. 174).” Sugiyama et al. [8] reviewing the studies since 2010, found that studies on the built environment and obesity produced inconsistent results—only 32 percent of the cross-sectional studies and 45 percent of the prospective (longitudinal) studies found an association. But, they also found that compactness was associated with less weight gain. They conclude that “[i]n order to address obesity through environmental initiatives, neighborhood environments may have to facilitate active living in multiple ways.” (no page number) Sturm and An [9] acknowledge that research on urban design and the built environment has produced mixed results that are sometimes contradictory, making it difficult to show a causal relationship.

Ding and Gebel [15] present an extensive and thorough review of the review papers on the built environment, physical activity, and obesity in order to identify research gaps and areas of improvement. Because this research area has produced such a large number of papers over the 2000s, there have also been a large number of review papers as well. Among the findings of their review was the need for longitudinal studies to minimize the impact of possible self-selection.

Plantinga and Bernell [11, 16] develop a theoretical model to explain the behavioral microfoundations of the observed association between obesity and sprawl. In their model, settlement type and physical activity level are jointly selected in equilibrium where “[r]esidents maximize utility by choosing lot size and calorie expenditure subject to their budget constraint” (2007, p. 860). The logic motivating Eid et al. [10] is similar, arguing that “someone who does not like to walk is both more likely to be obese and to prefer living where one can easily get around by car. For such individuals obesity is correlated with, but not caused by, the choice to live in a sprawling neighborhood. That is, we may observe more obesity in sprawling neighborhoods because individuals who have a propensity to be obese choose to live in these neighborhoods” (p. 386).

A recent challenge to the selection explanation is provided by Arcaya et al. [17] who used the relocation of Hurricane Katrina evacuees to test whether walkable environments are protective against weight gain. The fact that the New Orleans Hurricane Katrina survivors had little if any control over their relocation destination creates a natural experiment for studying urban sprawl and BMI. An argument of self-selection cannot be used in this situation, and so there is not an endogenity issue in looking at the built environment and BMI. The longitudinal data project that began in 2003, Resilience in Survivors of Katrina (RISK), collected pre- and post-hurricane data on parents from New Orleans. The authors found that those who were displaced and relocated to more sprawling areas, as defined by the county sprawl index, had higher BMIs, and the average weight increase was 5 percent. This finding makes a strong case that the built environment can facilitate individuals’ activity and affect weight.

Research examining the association between the built environment and walking or biking for transportation as a specific type of energy expenditure identify density, land use mix and proximity of non-residential destinations as strong correlates [18,19]. The literature on walking for transportation is more equivocal with respect to the importance of area attractiveness, personal safety or network connectivity [19]. The one comparative study of biking for transportation across US cities did find that bike infrastructure in the form of bike paths and bike lanes, the share of students in the population, and lower bike fatality rates were all positively associated with a higher bike commute share.

## Methods

### Data and Definitions

We used the publically-available 2006–08 American Time Use Survey (ATUS) and the Eating & Health Module (EH) data. The ATUS is a Bureau of Labor Statistics Survey, conducted by the Census Bureau, and the EH is a supplement developed and funded by USDA Economic Research Service. These data are available at http://bls.gov/tus/ and http://ers.usda.gov/Data/ATUS/. The ATUS User’s Guide (http://www.bls.gov/tus/atususersguide.pdf) discusses the extensive pre-testing and pilot testing that the Survey underwent before full production. The ATUS is a continuous survey that began in 2003, with interviews conducted nearly every day of the year. All respondents in the ATUS were previously in the Current Population Survey (CPS). A CPS panel is interviewed for 4 months, not interviewed for the following 8 months, then interviewed again for 4 months. The ATUS draws samples each month from those who have completed the 16-month CPS. The ATUS interview is 2–5 months after the final CPS month. Respondents are interviewed using a computer-assisted telephone-interviewing (CATI) system. The core of the survey is the 24-hour time diary covering 4am the day before the interview to 4am of the interview day.

One potential drawback of the ATUS diary data is that information on only one time-diary day per person is collected. There may be concern that some activities, such as engaging in sports and exercise, are not daily activities, and thus a one-day diary such as the ATUS is missing intrapersonal variability. However, some activities have a large degree of persistency, meaning that day-to-day variation is minimal. The American Association of State Highway and Transportation Officials found that commuting methods have a high degree of “brand loyalty” such that the “usual” mode was the same as the “actual” mode for a given day—driving alone, walking, and bicycling all had high shares of the usual mode being the actual mode on the survey day. For driving alone, 93.5 percent of commuters usually drove alone and also drove alone on the survey day; for those who walked to work, 80.2 percent; and for those who cycled to work, 73.0 percent, indicating persistency in these commuting modes [20]. Indeed, much of individuals’ daily activities can be classified as habitual repetition [21].

Nevertheless, food intake surveys are typically multiday surveys. For example, the NHANES, administered by Centers for Disease Control and Prevention, includes two 1-day food recall interviews (http://www.cdc.gov/nchs/nhanes.htm). However, because the second-day diaries have a higher rate of nonresponse, and because respondents’ consistent reports of less food consumption on the second day suggest under-reporting, some researchers elect to use only the first diary day [22].

Indeed, existing research supports using a one-day diary to analyze individuals’ activity patterns. Lambe et al. [23] examined food consumption using 14-day diaries in five locations in the European Union. Among their findings is that the quality of the diaries declined over the 14 days, with the best information and most variation obtained in the first three days. However, they found that mean intakes of a given food item were not affected by survey duration. More recently, Raux et al. [24] studied seven-day travel diaries for individuals in Ghent, Belgium and concluded that, while there is a large amount of interpersonal variability (differences across individuals in their travel patterns), there is small intrapersonal variability (variation across an individual’s seven days of time diaries). Likewise, Schmidt [25] studied seven-day diaries of Germans’ payments (consumer expenditures), cash and noncash, with a focus on cash payments, and found that survey fatigue is apparent and that more cash payments were recorded on day one. However, the distribution of payments on diary days 2–7 is similar, leading Schmidt to conclude “that additional diary days only increase the sample size, rather than provide additional information … (p.13).”

Another argument for using the ATUS 1-day time diary data is that the ATUS is a large and nationally representative, and so intrapersonal variability would not be an issue. Time diary data also has the advantage of being less subject to under- and over-reporting, including social desirability bias, than surveys that ask for the frequency of an activity or for estimates of time spent on specific activities [26 chapter 4,^27^]. Exercise in particular is an activity prone to over-reporting in questionnaire surveys [28].

We included only respondents who were employed, who engaged in paid work on their diary day (ATUS activity codes 05xxxx), and who performed some or all of their paid work activity at their workplace. We included only respondents age 20 and older in order to use the Centers for Disease Control (CDC) adult BMI categories (http://www.cdc.gov/healthyweight/assessing/bmi/adult_bmi/index.html). We used the ATUS Respondent, Activity, Summary Activity, and ATUS-CPS files, and the EH Respondent and Replicate Weights files. Every respondent in the ATUS was previously in the Current Population Survey (CPS) as the ATUS samples are drawn from the CPS panels’ final outrotations. We linked the ATUS files with the CPS files in order to extract geographical information. We used the BMI variable from the EH Module that is calculated from self-reported height and weight [29,30]. The resulting datafile contains 13,206 respondents.

We used the ATUS-Compendium of Activities bridge that was developed by Tudor-Locke et al. [31], MET bridge available at http://riskfactor.cancer.gov/tools/atus-met/] to examine the association between physical activity levels and compact settlement. This bridge assigns metabolic equivalent (MET) values to each of the over-400 ATUS activity codes, including the travel codes along with mode of transportation, and also to paid work activity by occupation. We use these codes to calculate a total MET value over the respondent’s diary day. For example, walking for recreation has a MET code of 3.80, so if the respondent reported engaging in walking for recreation for 15 minutes, then the MET value would be 3.8 times 15, which is 57.

Active commuters are identified as respondents who reported “biking” (TEWHERE = 17) or “walking” (TEWHERE = 14) as their transportation mode for “travel to work” (ATUS activities 180501 and 180589) or “travel to work-related activities” (180502). The advantage of using active commuting for examining health outcomes is its habitual nature. Active commuting is a small percent of all commuting modes, however someone reporting walking or biking to work on their diary day likely regularly walks or bikes to work. The habitual nature of commuting helps to overcome the fact that the ATUS collects only one diary day.

ATUS codes each leg of a trip by mode of transportation that the respondent reports, so biking or walking may be only one leg of the commute. ATUS codes travel activities as to their purpose, looking ahead to the next activity and location unless the next activity is at home in which case the travel purpose is coded according to the previous activity. As a result, this coding rule might undercount active commuters due to trip chaining; e.g., a trip to a coffee shop on the way to the workplace will not be coded as work-related travel but instead travel related to a food purchase (180782). This is more of a problem in calculating accurate commute times but may still result in an active commute undercount. However, we have no reason to think that those who, say, stop for coffee on their way to work or grocery shop on their way home are different with respect to urban form and BMI than others, so we expect no bias as a result. Alternatively, if respondents recall and report walking to their car in a large parking lot on their way to work then genuine active commuting might be over counted.

In order to observe a work commute, we include only respondents who engaged in paid work at their workplace on their diary day. In addition, we include only respondents living in metropolitan statistical areas since the CPS does not provide detailed geographic identifiers for respondents living in nonmetropolitan counties. The resulting data file contains 13,206 respondents of a total of 37,832 respondents in the 2006–08 ATUS-EH data. Of these, 12,405 are used in the analyses requiring self-reported height and weight needed to calculate BMI.

We use measures from Eid et al. [10] and Burchfield et al. [32], to examine contextual factors that differentiate compact from more sprawling settlement patterns. Most importantly, their measure of the degree of residential sprawl in urban land in 1992, developed in Burchfield et al., is defined as the percentage of undeveloped land in the square kilometer surrounding an average residential development, constructed using a grid of 8.7 billion 30 x 30 cells of remote sensing data. Other measures also adopted from Burchfield et al. include road density of major routes in the urban fringe, streetcar passengers per capita in 1902 indicating substantial compact urban development prior to mass production of the automobile, and two climatic variables (Heating Degree Days and Cooling Degree Days). From the ATUS-EH data we know if the respondent resided in the principal city of a metropolitan area or in the urban fringe. Population density of the Metropolitan Statistical Area (MSA) is computed from the 2000 Decennial Census [33].

### Regression Analysis Strategies

Because of the concern about self-selection, analysis is a multistep process to ensure that results are not driven by individuals’ unobservable characteristics. First we explicitly test the assumption that compactness is strongly associated with higher levels of physical activity. We then examine the association between compactness and active commuting. After demonstrating that overweight and obese workers engage in active commuting we examine the effect of active commuting on BMI as an endogenous treatment.

Examining the association between population density and energy expenditure is straightforward after controlling for possible confounding individual and contextual factors. If the association is driven by endogenous selection then an OLS specification is adequate as we are not interested in causality, that is, whether compact development compels physical activity. Confounding individual characteristics include age, gender, race/ethnicity, education level, presence of children in the household, and physical activity level of their primary occupation.

The other association of interest is that between compact development and active commuting. A logistic regression to examine mode choice (active commuting versus passive commuting) is valid as at this stage we are not interested in the causal question of whether compact development compels more active commuting.

Even if heterogeneous preferences for physical activity do not provide a plausible explanation for the general association between obesity and sprawl, it may nonetheless provide an explanation for behaviors of archetypal compact city residents that pursue active lifestyles. Thus, urban characteristics that increase the probability of active commuting but are not directly tied to compactness or sprawl may be important in identifying an unbiased effect; i.e., contextual variables which have a strong correlation with active commuting but are uncorrelated with BMI. Candidate variables that will be critical to the success of the endogenous treatment model discussed below are tested using the logistic regression model. Four candidate variables include (1) the MSA’s murder rate, (2) the college enrollment rate in the MSA among 18 to 24 year-olds, (3) the number of historical sites in the MSA, and (4) whether or not major cities within the MSA have received the Bicycle Friendly Community certification from the League of American Bicyclists.

Adverse weather conditions in the respondent’s MSA on the diary day is also examined as a possible factor that may be highly correlated with active commuting on the diary day, but uncorrelated with BMI. Detailed weather data from the North America Land Data Assimilation System Phase 2 [34] provided information on precipitation, temperature, humidity and wind speed for each respondents’ location on the diary day (National Centers for Environmental Prediction/Environmental Modeling Center 2012). Average wind speed above 6.2 mph proved to be the most useful weather variable with active commuting.

Justification for consideration as viable candidates is discussed below.

(1) Murder rate: A series of papers by Michael Sivak [35,36] identify a strong association between the murder rate and vehicle accident fatality rate in MSAs, an association that may be explained by the underlying level of aggressiveness. High levels of aggressiveness might dissuade otherwise willing active commuters, given the heightened level of vulnerability of those biking or walking. Accident data from Fatality Analysis Reporting (FARS) are available at http://www.nhtsa.gov/FARS. (2) College enrollment [33]: On a more positive note, “university cities” are often thought to be more conducive to active commuting with students providing the critical mass that encourages more walking or biking to work, measured by the enrollment rate of 18 to 24 year olds. (3) Historical sites (spreadsheet available at http://www.nps.gov/history/nr/research/): Workers might also be more likely to walk or bike to work if their surroundings are more aesthetically pleasing or more interesting. We use the number of historical sites in an MSA as an indicator of the built amenities that might induce more active commuting. It is possible that this variable is associated with earlier settlement and expansion which, like the streetcar passengers per capita in 1902 variable above, might be associated with more compact development. However, the strength of this association (and with body mass index) is an empirical question. (4) Bicycle friendly (available at http://www.bikeleague.org/content/communities): Active commuting might also be induced by the policies and infrastructure recognized by the League of American Bicyclists in their Bicycle Friendly Community certification. These include the existence of a bicycle master plan, bike lanes and multi-use paths, safety education for cyclists and drivers, active promotion of cycling in the community, enforcement of laws that make cycling safer, and the incorporation of cycling into evaluation and planning processes. Again, more compact cities may have a greater incentive to pursue certification but this too is an empirical question. Most importantly, the variable provides information on the effectiveness of local initiatives to promote active commuting.

Self-selection may be a very serious problem in trying to estimate the impact of active commuting on BMI when individuals who walk or bike to work are routinely characterized as particularly resolute and stalwart. Since those same personal characteristics which are unobserved are likely to have a large influence on BMI, the estimate of the impact of active commuting on BMI may owe more to these characteristics than engaging in the activity. If this were the case, then policy to promote active commuting as a way to reduce BMI in a population would not be effective.

The ideal way to control for this unobserved heterogeneity would be to use a longitudinal dataset. This is the estimation strategy that Eid et al. [10] and Plantinga and Bernell [11] use to examine the efficacy of promoting more compact settlement patterns to address the obesity epidemic. Unfortunately, the physical activity data included in the National Longitudinal Survey of Youth are too coarse and of questionable reliability to allow a direct test of their heterogeneous preferences assumption. With cross-sectional data such as ATUS-EH, a viable estimation strategy is to specify an endogenous treatment model where observable variables are used to predict the likelihood of the treatment, and the predicted value for the treatment variable is then used in the outcome regression. A strong negative correlation of the error terms from the treatment and outcome regressions would mean that unobservables associated with active commuting are also associated with lower BMI. Heterogeneous preferences or other unobserved personal characteristics would be presumed to play a large role in the effect of the treatment on the outcome. In the absence of a strong negative correlation of residuals, the parameter estimate on the predicted treatment variable are more easily interpreted as a valid average treatment effect as the observable predictor variables establish plausible counterfactuals. Since using compactness as a predictor of active commuting could proxy for unobserved preferences for physical activity we limit the geographic predictors to variables not associated with compactness.

In the specification of the endogenous treatment model we use the murder rate, adverse weather on the diary day, and bicycle friendly community certification as contextual predictors of active commuting. In addition, those personal characteristics that were most strongly associated with active commuting such as being African American, highly educated, poor, or the number of children in the household were also included in the treatment equation.

## Results

Table 1 provides a schematic overview of the successive steps that previews the findings. A detailed discussion of each step follows.

**Table 1:** Summary of Steps to Address Research Question.

Question	Are more compact developments conducive to more physical activity?
**Step 1**	a. Test assumption that compactness is associated with higher PA (as measured by total METs) and more moderate and vigorous activities.
	b. Found that share of principal city residents doing PA similar to others (Tables 2 and 3).
**Step 2**	a. Are residents of compact settlements more likely to engage in active lifestyles even though PA levels appear the same as for others?
	b. Determine research method—look at active commuting.
**Step 3**	a. Is there an association between compactness and active commuting, given that compactness provides the infrastructure for PA?
	b. Logit models of probability of active commuting—Table 4.
	c. Conclude: Compact settlement residents NOT more active, however place characteristics strongly associated with those that active commute.
**Step 4**	a. What is the association between BMI and active commuting?
	b. First, determine BMIs of active commuters—median active commuter is overweight but lower BMI than other commuters (Fig 1) → public health relevant.
	c. Next, quantile regressions results—active commute negatively and significantly associated with BMI, and larger impact at higher BMIs (Table 5).
**Step 5**	a. BMI and active commuting—what about endogeneity concerns, that is, self selection?
	b. Error term of active commuting regression is NOT strongly associated with error term from BMI regression.
	c. Endogenous treatment model results—active commuting is negatively and significantly related to BMI such that the active commuting effect is 11 fewer pounds (Table 6).
**Conclusion**	a. Promoting compact urban development may help lower BMIs and reduce the obesity epidemic by providing the infrastructure for more active lifestyles.
	b. Compact development of itself does not induce more PA.
	c. Promoting active commuting may be an effective way to increase PA and stem the obesity epidemic.

### Association between Compactness and Physical Activity

OLS regression results are presented in Table 2 for the association between metabolic equivalent minutes in the diary day and urban density and other confounding factors. Physical activities include all activities reported in the time diary which range from sleep to vigorous sports and other physical exercise. All estimates were calculated using SAS 9.3. Estimation procedures outlined in the *ATUS User’s Guide* (http://www.bls.gov/tus/atususersguide.pdf) and the EH Module *User’s Guide* (http://www.ers.usda.gov/media/138567/ap047_1_.pdf) were followed. Standard errors were calculated according to Section 7.5 of the ATUS User’s Guide. The EH Module Replicate Weights were used to calculate standard errors.

**Table 2:** Regression on Metabolic Equivalent Minutes in 1440 Minute Diary Day.

Parameter	Estimate	Standard Error	t Value	p-value	Mean
Dependent Variable				** **	2183.198
Intercept	2143.971	53.7332	39.9	< .0001	
Age	3.5601	1.764	2.02	0.0452	41.398
Age-Squared	-0.0366	0.0192	-1.9	0.0589	1877.938
Male	42.0568	6.3869	6.58	< .0001	0.541
African American	-46.0682	9.9662	-4.62	< .0001	0.116
Asian	-45.5717	17.1833	-2.65	0.0088	0.041
Hispanic	-13.2785	10.8344	-1.23	0.2222	0.153
Less than HS diploma	-56.5118	16.5871	-3.41	0.0008	0.090
Some College	0.6389	10.6229	0.06	0.9521	0.285
College Degree	-11.6082	9.9015	-1.17	0.2428	0.235
Advanced Degree	1.4294	12.0369	0.12	0.9056	0.129
HH # Children	31.3755	3.5918	8.74	< .0001	0.811
Occ. Stamina (0–6)	-1.8853	5.2626	-0.36	0.7206	1.115
Population Density	-7.0854	4.3622	-1.62	0.1063	-1.800
Principal City	-20.1502	7.4971	-2.69	0.008	0.311
Sprawl 1992	-0.8541	0.3904	-2.19	0.0301	40.362
Streetcar 1902	-0.0495	0.0369	-1.34	0.1816	110.949
Road Density	4.7276	11.9372	0.4	0.6926	0.924
Cooling Degree Days	-0.018	0.0099	-1.82	0.0699	1291.980
Heating Degree Days	-0.0038	0.0041	-0.92	0.3582	4455.704

N = 13,123 R^2^ = 0.0271 MSE = 304.37

Control group: Female, White, high school diploma.

Note: Only those who were age 20 and over, employed, engaged in paid work at their workplace on their diary day, and commuted to their workplace included.

Source: Authors’ estimates using data from 2006-08 American Time Use Survey and Eating & Health Module. Other data sources described in text.

The dataset used for estimation is restricted to workers commuting to work on their diary day, the same dataset that will be used to examine active commuting. The possibility that principal city residents expend less energy on work days but much more energy on non-work days was examined by constructing a dataset with all employed respondents. Forty-three percent of these respondents did not work on their diary day. The data confirmed that those not working on their diary day expended less energy than those commuting to work.

The low R-squared suggests that the personal and settlement characteristics do not do a very good job of explaining differences in activity levels. However, since differences in the preference for physical activity are assumed to be the primary reason for the observed association between obesity and sprawl, the negative and significant association (-20.1502, p-value = 0.008) between principal city residence and metabolic equivalent minutes contradicts expectations, indicating that principal city residence is associated with 20 *fewer* MET minutes on an average day. Although the association is negative and significant, 20 METs is less than one percent of the average daily METs.

In looking at metabolic minutes and the highest activity level observed during the diary day, we see that those who engage in high activity levels do not always have the most metabolic minutes. There are plausible explanations for why the compiled number of metabolic minutes may be lower for individuals who otherwise engage in more moderate or vigorous activity than more sedentary respondents. For example, the much shorter commute of principal city residents might allow them to sleep longer. We construct a frequency table to examine the highest metabolic equivalent activity in each respondent’s diary day for residents of principal or central cities and for residents of surrounding metropolitan counties. If principal city residents in fact have a preference for more physical activity, then a higher percentage of principal city residents should make up the Moderate (highest metabolic equivalent level of 5.9) or Vigorous (highest metabolic equivalent level of 8) activity categories. In fact, the share of residents in principal cities (53.79%) engaging in moderate or vigorous activity is very similar to the share of residents not in principal cities (53.02%) (Table 3).

**Table 3:** Frequency of Highest Activity Level Observed on Diary Day by Principal City Status (Weighted to Represent US Population).

	Sedentary	Light	Moderate	Vigorous	Total
Principal City	80,000	11,650,000	11,900,000	1,750,000	25,370,000
(Row%)	0.32%	45.90%	46.90%	6.89%	100%
Not Principal City	175,000	26,120,000	26,490,000	3,180,000	55,960,000
(Row %)	0.31%	46.68%	47.34%	5.68%	100%

Source: Authors’ estimates using data from 2006–08 American Time Use Survey and Eating & Health Module. Other data sources described in text.

We do not need to prove that residents of principal cities and more dense settlements expend less energy than peers in less dense metropolitan settlements. All that is required is evidence that principal city residents do not express a much stronger preference for physical activity that is central to behavioral explanations for the association between obesity and sprawl. Indeed, sprawl may have a larger impact on the other side of the energy balance equation by reducing the opportunity cost of food consumption through the greater prevalence of grocery supercenters [37]. Urban density is not a silver bullet for fighting the obesity epidemic not because physically active individuals have already self-selected compact settlements, but because compact settlements of themselves do not compel greater physical activity. Rather compact development may facilitate less automobile use and more physical activity for a select group who chose to do so [12]. If we view compact settlement as the requisite *infrastructure* for active living, then the argument turns to the more fundamental prior: Are residents of compact settlements more likely to engage in active lifestyles, even if relatively rare?

### Association between Compactness and Active Commuting

Of the 13,206 respondents included in the analysis, 606 (4.58%) reported walking or biking to work for at least one leg of their commute. The population-weighted share of active commuters is 4.78%, very close to the 4.89% active commute share for the 60 largest metro areas computed using the 2009 American Community Survey [38]. Population weighted estimates from ATUS suggest that residents of principal cities are more than twice as likely to walk or bike to work (8.25%) than residents of surrounding metro counties (3.21%).

Note that the consistently low share of active commuters helps explain why the topic has not been examined at the national level in the United States, at least for the adult population. More focus has been on active commuting by school children, since the share of students walking or biking to school has dropped dramatically over the last 30 years [39,40]. However, policy interest in the topic has increased markedly with the doubling of funding for pedestrian and biking transportation infrastructure since 2008, along with the announcement of a “policy sea change” in March 2010 at the Department of Transportation that gives biking and walking projects the same importance as automobiles in transportation planning and the selection of projects for federal money [41]. A recent study by the US Census Bureau identifies an increase of 60% of the active commuting mode share between the 2000 Decennial Census and pooled 2008–2012 American Community Survey Data [42].

Estimates from a logistical model of the probability of active commuting are provided in Table 4. Explanatory variables are the same as in the metabolic equivalents regression, but also include a variable indicating low income and five additional variables that may be highly correlated with active commuting but uncorrelated or weakly correlated with BMI. Socio-economic controls suggest a U-shaped relationship between status and active commuting. Those earning less than 185% of the poverty threshold income are much more likely to walk or bike to work, but this is also true of those with college and advanced degrees of all income levels. The age and gender variables are not significant in predicting active commuting, but African Americans are more likely to walk or bike to work, other things being equal. The number of children in the household was negatively associated with active commuting, suggesting that either time constraints or the greater necessity for trip chaining, such as dropping off or picking up children from schools or day care centers, may be an important factor in commute mode choice. Controls for season and year were not significant.

**Table 4:** Logistical Model of the Probability of Active Commuting.

	Long Regression (1)			Excl Historical (2)		Excl All Candidates (3)		
	Estimate	p-value	Odds Ratio	Estimate	p-value	Estimate	p-value	
Intercept	-3.9554	0.0005		-4.2904	0.0001	-3.359	0.0007	Intercept
Age	-0.0215	0.4971	0.979	-0.0241	0.4479	-0.0223	0.4832	Age
Age Squared	0.0002	0.6094	1	0.000218	0.5491	0.000195	0.5948	Age Squared
Male	0.1166	0.3577	1.124	0.1319	0.2988	0.1304	0.3014	Male
African American	0.5681	0.0015	1.765	0.5866	0.0009	0.5481	0.0012	African American
Asian	0.3662	0.1879	1.442	0.3523	0.2113	0.3899	0.1752	Asian
Hispanic	0.2593	0.1976	1.296	0.2696	0.1825	0.3139	0.1149	Hispanic
Less than HS	0.1925	0.5019	1.212	0.1744	0.5433	0.1667	0.563	Less than HS
Some College	0.0463	0.8107	1.047	0.0373	0.8458	0.0667	0.7262	Some College
College Degree	0.3964	0.0492	1.486	0.3916	0.0503	0.4067	0.0398	College Degree
Adv. Degree	0.4435	0.0405	1.558	0.4505	0.0366	0.4971	0.0215	Adv. Degree
Income < 185% Poverty	0.4769	0.0132	1.611	0.479	0.0127	0.4713	0.0133	Income < 185% Poverty
HH # Children	-0.1740	0.0036	0.84	-0.1701	0.0045	-0.1766	0.0033	HH # Children
Spring	0.0140	0.9358	1.014	0.0102	0.9535	0.0467	0.7866	Spring
Summer	0.1369	0.4536	1.147	0.1394	0.4416	0.1589	0.3715	Summer
Fall	0.0288	0.8871	1.029	0.0182	0.9281	0.0531	0.7896	Fall
2006	0.1454	0.5176	1.156	0.1444	0.5191	0.1316	0.559	2006
2007	-0.1078	0.6574	0.898	-0.1055	0.6621	-0.1032	0.669	2007
Sprawl 1992	0.0042	0.6555	1.004	0.00909	0.3146	0.00461	0.594	Sprawl 1992
Streetcar Pass 1902	0.0000	0.9808	1	0.000862	0.2325	0.00112	0.0882	Streetcar Pass 1902
Road Dens Fringe	-0.3107	0.1812	0.733	-0.0793	0.7391	-0.138	0.5708	Road Dens Fringe
Cooling Deg Days	-0.0001	0.6388	1	0.000012	0.942	-0.00014	0.3822	Cooling Deg Days
Heating Deg Days	0.0001	0.1255	1	0.000131	0.0266	0.000122	0.0393	Heating Deg Days
Principal City	0.8217	< .0001	2.274	0.8235	< .0001	0.8259	< .0001	Principal City
Pop. Density	0.2198	0.0710	1.246	0.3561	0.001	0.3352	0.0008	Pop. Density
Murder Rate 2006	-0.0378	0.1543	0.963	-0.0431	0.1018			Murder Rate 2006
Coll. Enr 18–24	0.0208	0.0521	1.021	0.0209	0.0491			Coll. Enr 18–24
Historical Sites	0.0131	0.0010	1.013					Historical Sites
Bike Fr. Comm.	0.1944	0.1615	1.215	0.3074	0.0178			Bike Fr. Comm.
Wind >6.2mph	-0.6766	0.0975	0.508	-0.6365	0.1141			Wind > 6.2mph

Control Group: Female, White, high school diploma, Winter, and 2008. Note: Only those who were age 20 and over, employed, engaged in paid work at their workplace on their diary day, and commuted to their workplace included. Source: Authors’ estimates using data from 2006–08 American Time Use Survey and Eating & Health Module. Other data sources described in text.

The examination of urban characteristics uses a long and short regression strategy to 1) identify those characteristics which appear to be most salient in explaining active commuting, and 2) to help identify valid contextual variables for examining the relationship between active commuting and BMI. Residents in principal cities are more likely to be active commuters but population density was not significant, at least not at the 0.05 level. Of the four contextual variable candidates, only the number of historical sites is significant at the 0.05 level.

Note that the p-values in this analysis on the metropolitan variables should be viewed with caution. Valid standard errors of estimates for variables with clustered values over individual observations require a cluster robust variance-covariance estimator. Unfortunately, it is not possible to estimate a system using both the replicate weights that ensure valid variance estimates from a nonrandom sample and a cluster robust VCE. The results reported here use only replicate weights. Separate estimations of the model without replicate weights compared the cluster robust VCE standard errors to conventional standard errors. The differences were small and in this case largely inconsequential regarding inference. For example, the p-values for Murder Rate 2006, College Enrollment 18–24 and Historical Sites went from 0.1539, 0.0679 and 0.0018, respectively, using conventional estimates of standard errors to 0.137, 0.080 and 0.029 using a cluster robust VCE.

The possibility that the contextual variable candidates themselves are proxies for compactness is investigated in two short regressions (Table 4 equations (2) and (3)). We first estimate the model excluding the number of historical sites in an MSA. The estimates suggest that the historical sites variable is a powerful proxy for compactness since the population density variable is now significant. The exclusion of the historical sites variable also increases the precision of the college enrollment and bicycle friendly community coefficient estimates so they are now significant at the 0.05 level. If we exclude all of the candidate contextual variables, the estimates on the variables representing compactness do not change substantially, suggesting at most a weak relationship between the remaining three candidates and compactness.

Of the candidate contextual variables, the magnitude of the Wind > 6.2 mph indicator variable suggests that active commuting is half as likely on these very windy days with an odds ratio of 0. 508, albeit only marginally significant at the 0.0975 level. Historical sites in an MSA provides the most precise estimate, capturing aspects of the age of settlement predating the advent of the automobile along with the quality of the built environment. In terms of magnitude, residents of Boston, Massachusetts would be 68% more likely to walk or bike to work than residents of Orlando, Florida. (Estimates calculated from logit regression results in Table 4, calculations not shown.). But residents of Boston would only be 29% more likely to walk or bike to work than their Orlando peers given the relative shares of enrollment of college age residents (not shown).

To this point we have demonstrated that while residents of compact settlements generally are not more physically active than peers in more sprawling settlements (regression model results), place characteristics are strongly associated with the small share of residents that walk or bike to work (logistical model results). Next we look at the association between active commuting and BMI.

### Association between BMI and Active Commuting as an Endogenous Treatment

Before moving onto the results from the endogenous treatment model it is important to demonstrate that active commuting is a viable choice for overweight and obese individuals. Even a dramatic reduction in BMI attributable to active commuting would have little relevance for public health initiatives if these effects were limited to the normal weight population.

**Fig 1:**
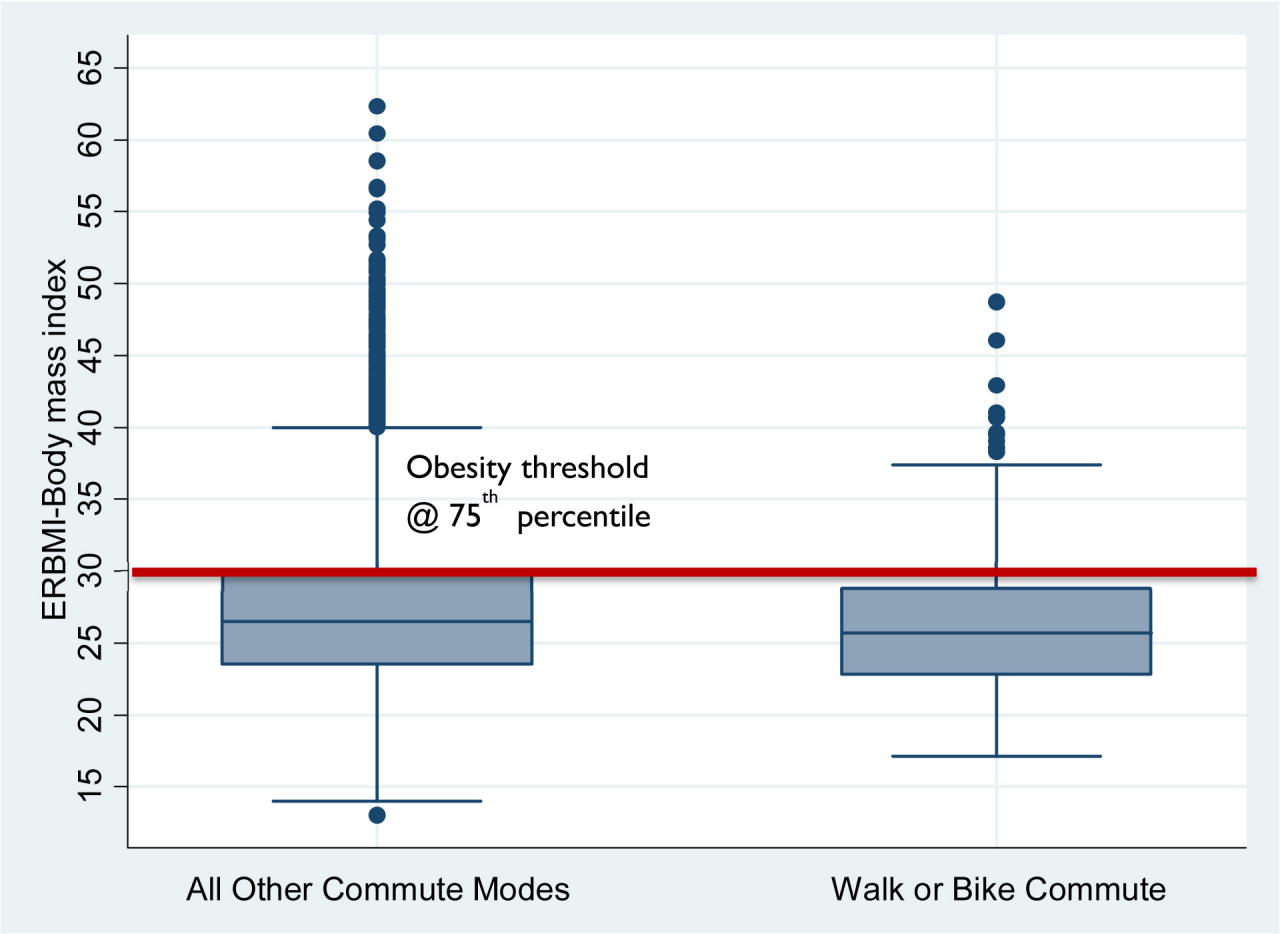
Boxplot of BMI by Active Commuting Status. Source: Authors’ estimates using data from 2006–08 American Time Use Survey and Eating & Health Module.

Fig 1 provides a boxplot of the BMI distribution by active commute status. The active commuting distribution is tighter with many fewer underweight and extremely obese respondents. But the most telling statistic is the median which confirms that the representative commuter is overweight (BMI great than 25) whether they are active commuters or not. The fact that the majority of active commuters are overweight or obese confirms that this strategy for weight control is applicable to that portion of the population that needs it most. The boxplot comparison is suggestive that active commuting is associated with weight reduction but this comparison does not control for confounding factors.

Table 5 provides a summary of results from a number of quantile regressions estimated simultaneously at relevant percentiles that controls for confounding factors and allows a precise estimate of the magnitude of weight loss associated with active commuting. The clear pattern that emerges is an increasing effect of physical activity on BMI, either in the form of active commuting or more strenuous work, and an increasing effect of more compact urban form on BMI at higher levels of BMI. The effect of active commuting on BMI is nearly four times larger in the 90^th^ quantile regression (-2.3234) relative to the 11^th^ quantile (-0.6623, corresponding with the middle of the normal weight range or a BMI of 21.75). These results are reinforced by a similar increase in magnitude with respect to the requirement for occupational stamina between the 11^th^ (-0.0991) and 90^th^ quantile (-0.4179) The stamina measure was derived from O*Net, produced by the Employment and Training Administration of the Department of Labor, and available at http://www.onetonline.org/. Both active commuting and occupational stamina represent moderate intensity activity on a regular basis, providing strong empirical support for the approach in the proposed *Dietary Guidelines for Americans* [43] to increase physical activity getting to work and at work to thwart obesity in the population.

**Table 5:** Parameter Estimates from Simultaneous Quantile Regression on Body Mass Index.

	11th Quantile Midpoint Normal Weight Category BMI = 21.75		50th Quantile Median BMI = 26.5		75th Quantile Approximate Obesity Threshold BMI = 30.1		90th Quantile Approximate Class II Obesity Threshold BMI = 34.3	
	Estimate	p-value	Estimate	p-value	Estimate	p-value	Estimate	p-value
Intercept	16.5072	<0.001	20.1132	<0.001	23.9933	<0.001	29.8325	<0.001
Age	0.1739	<0.001	0.2073	<0.001	0.2418	<0.001	0.2869	<0.001
Age Squared	-0.0015	<0.001	-0.0018	<0.001	-0.0024	<0.001	-0.0032	<0.001
Male	2.2604	<0.001	1.9247	<0.001	1.2546	<0.001	-0.0304	0.907
African American	0.9824	<0.001	2.2015	<0.001	2.5577	<0.001	2.4602	<0.001
Asian	-1.1128	<0.001	-1.5685	<0.001	-2.1043	<0.001	-2.5275	<0.001
Hispanic	0.4069	0.003	0.8126	<0.001	0.7809	0.001	0.2453	0.505
Less than HS	-0.2120	0.348	-0.1160	0.646	-0.3475	0.278	-0.2518	0.599
Some College	-0.0778	0.574	-0.1020	0.564	-0.1073	0.63	0.0226	0.951
College Degree	-0.5117	<0.001	-1.0095	<0.001	-1.3404	<0.001	-1.9569	<0.001
Adv. Degree	-0.7300	<0.001	-1.5540	<0.001	-2.0247	<0.001	-2.7762	<0.001
Inc < 185% Poverty	-0.0137	0.922	0.6707	<0.001	0.8627	<0.001	1.1996	0.001
HH # Children	0.0939	0.043	0.0872	0.101	-0.0067	0.927	-0.2100	0.109
Creative Occ. (0–1)	-0.2649	0.149	-0.2903	0.099	-0.7436	0.01	-0.1542	0.732
Occ. Stamina (0–4)	-0.0991	0.148	-0.1879	0.003	-0.3742	<0.001	-0.4179	0.008
Spring	0.0307	0.815	-0.0607	0.675	0.0236	0.912	0.2223	0.553
Summer	0.0838	0.469	-0.0531	0.697	-0.0803	0.689	0.1025	0.753
Fall	0.0650	0.641	0.0193	0.897	-0.0854	0.67	-0.1685	0.605
2006	-0.0829	0.596	-0.0864	0.677	-0.4496	0.061	-1.2997	0.016
2007	-0.0054	0.973	-0.1860	0.371	-0.2135	0.362	-1.1632	0.032
Principal City	-0.2236	0.024	-0.2224	0.046	-0.4525	0.01	-0.1007	0.737
Pop. Density	-0.0110	0.804	-0.2231	<0.001	-0.3788	<0.001	-0.3211	0.007
Active Commute	-0.6623	<0.001	-0.6490	0.012	-0.7426	0.013	-2.3234	<0.001
Pseudo R^2^		0.075		0.0513		0.0359		0.0425
N =		12,405		12,405		12,405		12,405

Control Group: Female, White, high school diploma, Winter, and 2008. Note: Only those who were age 20 and over, employed, engaged in paid work at their workplace on their diary day, and commuted to their workplace included. Source: Authors’ estimates using data from 2006–08 American Time Use Survey and Eating & Health Module. Other data sources described in text.

Finally, the endogenous treatment model in Table 6 provides information on whether the reductions in BMI associated with active commuting are directly attributable to this activity or if active commuting is merely correlated with unobservable factors that are the true cause of the reduction. First, a model of the probability of active commuting is estimated, and then those estimates are used for the active commuting right-hand-side variable in the BMI model. The specification of the outcome equation is identical to the quantile regressions (Table 5), with the important difference that active commuting is now its predicted value. The predictors of active commuting with the exception of the adverse weather variable are all significant and contextual variables are excluded from the outcome regression to ensure the average treatment effect is identified. The magnitude of the active commuting estimate (-1.83) is about three times as large in absolute value as in the median quantile regression from Table 5 (-0.65). An average treatment effect of -1.83 from Active Commuting on BMI translates into almost 11 fewer pounds for the average respondent who walks or bikes to work. Most importantly, the correlation of the error terms between the treatment and outcome equation was weakly positive (rho = 0.0781), dismissing concerns that the observed negative association between active commuting and BMI is explained by unobserved characteristics.

**Table 6:** Endogenous Treatment Effects Model: BMI Outcome Regression, Active Commute Treatment Regression.

BMI	Estimate	Std. Err.	z	p-value	[95% Conf. Interval]	
Age	0.2581	0.0342	7.55	<0.001	0.1911	0.3251
Age Squared	-0.0026	0.0004	-6.86	<0.001	-0.0033	-0.0018
Male	1.4713	0.1369	10.75	<0.001	1.2031	1.7395
African American	1.9709	0.2191	9	<0.001	1.5416	2.4003
Asian	-1.8656	0.3282	-5.68	<0.001	-2.5089	-1.2224
Hispanic	0.7836	0.2058	3.81	<0.001	0.3803	1.1869
Less than HS	-0.4442	0.2865	-1.55	0.121	-1.0058	0.1174
Some College	-0.2205	0.1990	-1.11	0.268	-0.6106	0.1696
College Degree	-1.3823	0.1954	-7.08	<0.001	-1.7652	-0.9994
Adv. Degree	-1.9537	0.2146	-9.1	<0.001	-2.3743	-1.5331
Income < 185% Poverty	0.2913	0.1909	1.53	0.127	-0.0828	0.6653
HH # Children	-0.0061	0.0620	-0.1	0.921	-0.1277	0.1154
Highly Creative Occ.	-0.2021	0.2545	-0.79	0.427	-0.7009	0.2966
Occ. Stamina (0–6)	-0.2371	0.0894	-2.65	0.008	-0.4124	-0.0618
Spring	0.2166	0.1855	1.17	0.243	-0.1469	0.5801
Summer	-0.0492	0.1798	-0.27	0.784	-0.4016	0.3032
Fall	-0.0665	0.1850	-0.36	0.719	-0.4290	0.2961
2006	-0.3929	0.2332	-1.68	0.092	-0.8499	0.0642
2007	-0.3357	0.2348	-1.43	0.153	-0.7959	0.1246
Principal City	-0.2236	0.1426	-1.57	0.117	-0.5032	0.0559
Pop. Density	-0.1992	0.0688	-2.89	0.004	-0.3341	-0.0642
*Active Commute*	-1.8302	0.6869	-2.66	0.008	-3.1764	-0.4840
Intercept	21.3217	0.8255	25.83	<0.001	19.7038	22.9397
*Active Commute*						
Black	0.3522	0.0771	4.57	<0.001	0.2011	0.5033
College Degree	0.2217	0.0711	3.12	0.002	0.0823	0.3610
Adv. Degree	0.2824	0.0796	3.55	<0.001	0.1263	0.4384
Inc < 185% Poverty	0.3010	0.0744	4.05	<0.001	0.1553	0.4468
HH # Children	-0.0634	0.0261	-2.43	0.015	-0.1144	-0.0123
Murder Rate 2006	-0.0213	0.0100	-2.12	0.034	-0.0409	-0.0016
Bike Friendly Comm.	0.3385	0.0586	5.77	<0.001	0.2236	0.4534
Wind > 6.2 mph	-0.1297	0.1838	-0.71	0.481	-0.4899	0.2306
Intercept	-1.8866	0.0794	-23.77	<0.001	-2.0422	-1.7311
rho	0.0781	0.0468			-0.0141	0.1690
sigma	5.2814	0.0668			5.1521	5.4140
lambda	0.4127	0.2474			-0.0723	0.8977
LR test of indep. eqns. (rho = 0): chi2(1) = 2.76 Prob > chi2 = 0.0966						

Control Group: Female, White, high school diploma, Winter, and 2008 Note: Only those who were age 20 and over, and employed included. Source: Authors’ estimates using data from 2006–08 American Time Use Survey and Eating & Health Module.

Separate OLS regressions on the active commuting and non-active commuting subsamples were estimated as a robustness check and to assess whether the model assumption of no interactions between treatment and independent regressors was appropriate. The difference in the OLS intercept was -1.83 (19.48 for active commuters and 21.31 for non-active commuters) or nearly identical to the endogenous treatment point estimate and the slope coefficient estimates were similar.

## Discussion

The argument that promoting compact urban development might help stem the obesity epidemic had been challenged by others’ evidence consistent with more physically active people self-selecting such environments [10,11]. Their analyses effectively demonstrate that compact development does not induce physical activity but rather might enable physical activity for those so predisposed. The policy implications of this conclusion are seemingly inert since the desirable outcome of increased physical activity is purportedly baked into individual preferences, so only those who are predisposed to physical activity engage in it.

To test this interpretation, that compact urban development is not effective, we used the 2006–2008 American Time Use Survey and the Eating & Health Module data to explicitly examine physical activity levels of respondents living in compact and in more sprawling metropolitan areas. Our findings do not support the hypothesis that physical activity is generally higher for people living in principal cities and more densely populated urban areas, consistent with other analyses that have failed to detect an association between physical activity and residential ‘walkability’ [6,7]. Instead, our findings support the hypothesis that more compact development enables active commuting for a small percentage of normal weight, overweight, and obese residents. Empirically, the association between active commuting and reduction in BMI is substantial. So, few people in compact development areas engage in physical activity, but those who do so benefit from it with lower BMIs, and they are not necessarily individuals who would be predisposed to physical activity. This means that infrastructure can indeed be effective.

### Strengths and Limitations

The biggest advantage the ATUS provides in assessing the association between physical activity and compactness is its broad coverage. Negative findings are easily dismissed if the results are not generalizable to the population of interest (limited coverage of the sample), or if the phenomenon of interest is too narrowly defined (limited coverage of the variable). The comprehensive geographic coverage of an extensive set of activities in the ATUS addresses both concerns: the sample is representative of the US population and the activity lexicon contains more than 400 activities over 17 major activity groups. Another advantage of using time diary data is that time diaries are considered a neutral method of collecting data on time spent in various activities. They are less subject to under- and over-reporting including social desirability bias than surveys that ask for frequency of an activity or for estimates of time spent on specific activities [26,27]. In particular, self-reported exercise and activity levels have been shown to be prone to over-reporting in response to direct survey questions on these activities [44,45], making the time diary data desirable for exercise and physical activity research.

One possible criticism of the data for comparing activity levels across settlement types is the impossibility of minute detail in the collection of different activities: the average number of diary activities reported by each individual is about 20, and only a handful of respondents report more than 70 activities in any single survey year. Respondents usually report activities that lasted 5 minutes or longer. Thus, very brief activities that might differ in energy expenditure across settlement types will not be recorded, such as walking up a flight of stairs. Survey collection methods studies have found that one-day diaries do well at capturing what an individual did the day before so this criticism could only be confirmed by assessing differences in physical activity using accelerometers. There may be concern that some activities are not daily activities and thus that a one-day diary such as the ATUS lacks intrapersonal variability. However, some activities, such as eating patterns, have a large degree of persistency, meaning that day-to-day variation is minimal; Wansink’s [46] *Mindless Eating* discusses the myriad external influences that result in eating habits. Exercise is also considered to be a habit, and researchers have studied what contributes to habitual exercise [47,48]. Indeed, much of an individual’s daily activities can be classified as habitual repetition [49]. Another argument for using the ATUS one-day time diary data is that the ATUS are large and nationally representative, and so intrapersonal variability would not be an issue.

A significant limitation plaguing many studies is the summary city or metropolitan area variables that may misrepresent the built environment where active commuting decisions are actually made [50]. Because our data only allow us to geocode respondents by metropolitan statistical area, and whether the respondent resides in the principal city, our analysis is also subject to this limitation.

### Implications of Similar Physical Activity Levels across Settlement Types

The association in the literature between more compact development and lower BMI has provided strong support for the assumption that residents of more compact cities tend to be more physically active and self-select walkable areas. Our analysis of a large nationally representative sample that includes an inclusive set of activities does not support this assumption. Alternative explanations of the negative association between compactness and BMI related to food consumption patterns deriving from food buying habits are reinforced by our negative findings [28].

These negative findings compel a more sober view of the potential role of compact development in addressing the obesity epidemic. Compact development does not inevitably induce more physical activity. Rather, compact development provides an infrastructure that may enable more active lifestyles. Given the possible limitations of the ATUS 24-hour recall diary, the active lifestyle dimension we focus on is active commuting; i.e., walking or biking to work, as a persistent, habitual activity. Our findings, from the first nationally representative model of active commuting in the United States suggest that active commuting—through practiced by a small percent of those employed—is more prevalent in more compact and more densely populated metropolitan areas.

### Implications of the Magnitude of the Effect of Active Commuting on BMI

The critical question is whether the impact of active commuting on body mass index is large enough to justify public efforts promoting it. Quantile regressions demonstrate that the magnitude of negative association between active commuting and BMI increases in absolute value in progressing from normal weight to obese individuals. Under the assumption of exogeneity, active commuting would appear to be a particularly effective lifestyle strategy for thwarting the onset of obesity.

### Implications of the Effect on BMI Controlling for Endogeneity

The potential efficacy of promoting active commuting as a strategy to combat obesity was more fully investigated by correcting for the endogeneity of active commute mode choice. An endogenous treatment model was developed, using the MSA murder rate, adverse weather conditions and whether major cities in an MSA had received a Bicycle Friendly Community certification from the League of American Bicyclists, to examine whether it is the activity or the unobservable characteristics explaining engagement in the activity that is responsible for reductions in BMI. The analysis finds that the strong negative association between active commuting and BMI is *not* explained by unobserved factors meaning that predisposition to physical exercise or self-selection to walkable areas was not a significant factor in explaining the lower BMIs. This result bodes well for the efficacy of the promotion of walking or biking to work as a way to stem the obesity epidemic.

These findings support health recommendations that assert that small changes, either small reductions in calories or small increases in activity level, can make a big difference over time. In particular, the NIH suggestion to “Get off the bus a stop early, and walk” for physical activity (http://www.nhlbi.nih.gov/health/public/heart/obesity/wecan/get-active/getting-active.htm) may indeed be a useful strategy for individuals. It also suggests that those too busy to set aside daily time for exercise as an activity, might instead successfully use their commute, either all or part of their travel to work, to increase their activity levels.
